# Epidemiology of rotavirus diarrhea in children under 5 years in Northern Cameroon

**Published:** 2012-04-17

**Authors:** Valentine Ngum Ndze, Achidi Eric Akum, Gonsu Hortense Kamga, Lyonga Emilia Enjema, Mathew Dioh Esona, Krisztian Banyai, Obama Abena Marie Therese

**Affiliations:** 1 Faculty of Medicine and Biomedical Sciences, University of Yaoundé I, Cameroon; 2 Faculty of Health Sciences, University of Buea, Cameroon; 3 Division of Viral Diseases, National Center for Immunization and Respiratory Diseases, Centers for Disease Control and Prevention, Atlanta, GA, USA; 4 Veterinary Medical Research Institute, Hungarian Academy of Sciences, Hungariat krt. 21, Budapest 1143, Hungary

**Keywords:** Rotavirus, diarrhea, Cameroon, children, epidemiology, prevalence

## Abstract

**Background:**

Rotavirus still remains the major cause of diarrhea in children below 5 years. No data on rotavirus epidemiology is available in the Northern regions of Cameroon. We aimed to determine the prevalence of group A rotavirus (RVA) in children below 5 years with diarrhea in two regions of Northern Cameroon (North West and Far North Regions) so as to improve our knowledge on the burden of rotavirus disease for imminent introduction of a rotavirus vaccine.

**Methods:**

Stool samples were collected during 2010 and 2011 from 390 children below 5 years presenting with diarrhea in four hospitals in Northern Cameroon and were screened for rotavirus group A by reverse transcription-polymerase chain reaction.

**Results:**

This study revealed that 42.8% of the children below 5 years had group A rotavirus infection, 46.5% in the Far North region while the North West had a prevalence of 33.9%. Of the 252 hospitalized and the 138 outpatient children, 124(49.2%) and 43(31.2%) (P=0.00085), respectively, were positive for group A rotavirus. Children below 24 months were most affected (44.7%), while the age group 49-60 months had the lowest prevalence (25%). The RVA prevalence was 44.6% in the urban and 28.9% in the rural settings of our study. It was observed that the proportion of children with diarrhea who had rotavirus accompanied with fever and vomiting in the outpatient group and inpatient group were 13.0% and 28.6% respectively, P=0.03.

**Conclusion:**

This study showed high incidence of rotavirus infection especially among hospitalized children in Northern Cameroon, suggesting that rotavirus is a major cause of childhood morbidity and mortality in this area.

## Background

Diarrhea still remains one of the leading causes of childhood mortality worldwide particularly in developing countries, causing approximately 1.87 million deaths each year [1]. Group A rotavirus (RVA) infection is the most important cause of severe diarrhea in childhood causing 20% of fatal diarrhea in young children [2, 3]. The annual estimates of rotavirus-related mortality in children 4].

Since 2006, two rotavirus vaccines, RotaTeq^TM^ (Merck and Co, PA, USA) and Rotarix^®^ (GlaxoSmith Kline Biologicals, Rixensart, Belgium), have been licensed, and they are recommended for use in all countries by WHO, particularly in those countries with high diarrhea-related mortality in children younger than 5 years [5]. Limited data is available on rotavirus disease burden in Cameroon, particularly in the North West and Far North regions. The aim of this study was to determine the prevalence of group A rotavirus in children below 5 years with acute diarrhea in two regions of Northern Cameroon (North West and Far North Regions) so as to improve our knowledge on the burden of rotavirus disease in Cameroon for imminent introduction of a rotavirus vaccine.

## Methods

### Study site and study population

A descriptive cross-sectional study was conducted from October 2010 to July 2011 in Northern Cameroon (at the Regional Hospital Maroua and at the Domayo Djama integreted health center in the Far North region; and at the Regional hospital Bamenda and at the Esu integrated health centre in the North West region). The Far North region vegetation is mainly the Sahel type and is dry all year round with minor rains in the months of June, July and August. The North West region is mainly grassland characterized by equal dry chilly and rainy seasons. The population is urban (Maroua and Bamenda) and rural (Esu) and the major occupation in the study area is subsistence agriculture supplemented with fishing and dairy farming in the Far North region. Included in the study were all in- and out-patients less than 5 years who presented with acute diarrhea at the study sites and whose parents gave their informed consent to participate in the study.

### Sampling and Sample collection

Participants were recruited in the order at which the cases presented. After informed consent was obtained, a pediatrician, medical doctor or consulting nurse assigned to the study examined each patient and filled out the demographic data and information on clinical symptoms and illness onset on a standardized questionnaire. A single fresh stool per participant per diarrhea episode was collected following microbiologically approved techniques. The samples were transported to the Hospital laboratory within 2 hours after collection and stored at -20°C and later transported on dry ice to the Veterinary Medical Research Institute, Hungarian Academy of Science, Budapest-Hungary where rotavirus detection was carried out.

### Laboratory detection of RVA

Fecal suspensions (10%) were prepared in phosphate buffered saline and stored at -20°C. Rotavirus dsRNA was extracted from 100µl stool suspension using the Promega kit by the Cobett Nucleic acid automatic extractor following the manufacturer's instructions [6]. Rotavirus A was detected by one-step RT-PCR (QIAGEN Kit) of gene 6 with VP6F and VP6R primers previously described [7]. In brief, denaturation of dsRNA was carried out at 97°C for 5 minutes, then reverse transcription carried out at 52°C for 30 minutes, activation of Taq polymerase at 95°C for 15 minutes followed by 40 cycles of PCR (denaturing at 95°C for 30sec, annealing at 52°C for 30sec, and extension at 72°C for 1 min) and a final extension cycle (72°C for 3 min). The cDNA was then ran on 1% Agarose gel (Seakem^®^ LE agarose) electrophoresis in standard Tris-Borate-EDTA buffer to determine samples positive for group A rotavirus. Gels were stained with Gelred (Biotum).

### Data analysis

Data was analyzed using Microsoft excel and Epi Info version 3.5.1. Prevalence was expressed as percentages. The Chi square test was used to analyze categorical variables and the Yates correction test where appropriate. A 95% confidence interval (CI) was calculated and values of P < 0.05 were considered significant.

### Ethical issues

An ethical clearance was obtained from the Cameroon National Ethics Committee and administrative authorizations obtained from the different health care settings in our study.

## Results

**Figure 1:**
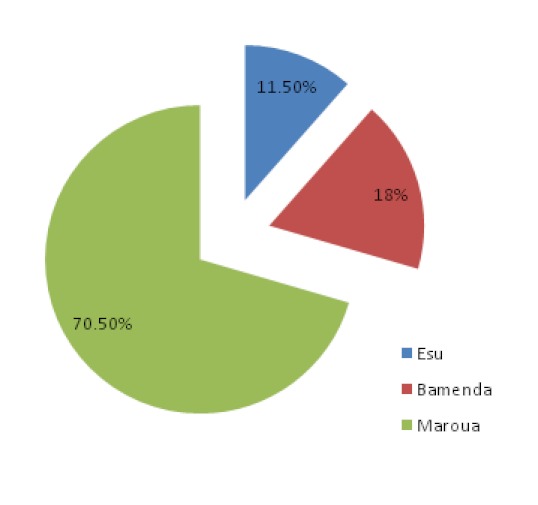
Proportion of samples collected from the different study sites

A total of 390 infants and young children with diarrhea were included in this study, 275 (70.5%) from Maroua, 70 (18%) from Bamenda and 45 (11.5%) from Esu (Figure 1) with a mean age of 14.8 months (median, 11 months; range 1-60 months). The sex distribution was 179 (45.9%) females to 211 (54.1%) males with mean ages of 15 months and 14.7 months (median, 11 months and 12 months, respectively). Of the 390 children with diarrhea, 252 (64.6%) were hospitalized and 138 (35.4%) were of the out-patient department.

This study reveals that 42.8% (167/390) of the children presenting with diarrhea were infected with group A rotavirus. The prevalence of RVA in children in the in-patient (49.2%) and the out-patient (31.2%) was significantly different, *X* ^*2*^(Yates) =11.14, P=0.00085. The prevalence of group A rotavirus in the two sexes was similar 40.8% in males as to 45.3% in females as shown in Table 1. There was no significant difference observed when the prevalence of rotavirus in males was compared to that of females, *X* ^*2*^ (Yates) =0.43, P=0.43. The highest number of cases with rotavirus infection was observed in the age group 0-12 months (47.9%) while the age group 49-60 months had the lowest prevalence (25%) (Table 1).

**Table 1:** Sociodemographic Characteristics

Variable	Categories	Total	Positive n(%)	P-value
**Gender**	Male	211	86 (40.8)	0.43
	Female	179	81 (45.3)	
**Age group (months)**	<12	217	104 (47.9)	
	13-24	121	47 (38.8)	
	25-36	30	10 (33.3)	
	37-48	14	4 (28.6)	
	49-60	08	2 (25.0)	
**Health setting**	RHB	70	26 (37.1)	0.049
	RHM	237	109 (46.0)	
	EIHC	45	13 (28.9)	
	DIHC	38	19 (50.0)	
**Nature of Site**	Urban	345	154 (44.6)	
	Rural	45	13 (28.9)	

n= number of RVA positive cases. RHB: Regional hospital Bamenda, RHM: Regional Hospital Maroua, EIHC: Esu integrated health centre, DIHC: Domayo Djama integrated health center.

It was observed that children positive for rotavirus who had diarrhea accompanied with fever and vomiting in the outpatient group and inpatient group were 13.0% and 28.6% respectively, P=0.03, (Table 2).

**Table 2:** Clinical characteristics in relation to presence of RVA in hospitalized and out-patient cases

Clinical symptom		Rotavirus Positive n (%)		
		In-patient	Out-patient	P-value
Fever	Yes	88 (54.3)	28 (35.4)	0.04
	No	36 (40.0)	15 (25.4)	
Vomiting	Yes	96 (64.4)	23 (43.4)	0.049
	No	28 (27.2)	20 (23.5)	
Fever +Vomiting		72 (28.6)	18 (13.0)	0.007

P-value 0.03

## Discussion

The aim of this study was to determine the prevalence of rotavirus in children ≤ 5 years presenting with acute gastroenteritis in two regions of Northern Cameroon (North West and Far North Regions). Our study showed a rotavirus prevalence of 42.8% (North West region 33.9% and Far North region 46.5%) and was significantly higher among hospitalized children.

**Figure 2:**
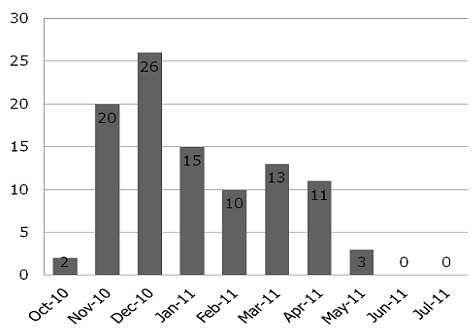
Monthly (seasonal) prevalence of group A rotavirus in Northern Cameroon

Many studies have shown the important role of rotavirus as a cause of diarrhea in children in both developed and developing countries [8–13]. Most of the cases occurred in children less than 5 years of age. This detection rate is greater than what was obtained by Esona and co-workers (21.9%) during a 2003 study in Western Cameroon but similar to what was found in recent studies conducted in other parts of Africa [4, 14, 15]. It is noteworthy that detection of rotavirus in this study was done by RT-PCR while that of the 2003 study in Western Cameroon was done using an enzyme immunoassay (EIA) method. The Far North region is a flood prone region and has the highest occurrence of diarrhea disease in Cameroon. These factors combined might explain the difference in the prevalence estimates of rotavirus observed in Cameroon. The prevalence of rotavirus in the Far North region was 46.5% (128/275) indicating that rotavirus is an important contributor to the causes of diarrhea in this region of Cameroon and rotavirus may have aggravated the cholera situation since most of the rotavirus cases identified in this study site were recorded just after the cholera epidemic. There was a significant difference in the prevalence of rotavirus between the health care settings with higher incidence among hospitalized children. It should be noted that the sampling was biased and therefore not sufficiently comparable. The prevalence of rotavirus in the rural area (28.9%) was lower than that in the urban area (44.6%). The fact that patients from rural area had access primarily to outpatient clinic could have distorted this finding. Also the rural population may only report to the hospital when diarrhea had already subsided or toward the end of the episode. All this is due to transportation and availability of over the counter medication which may mask the disease. Cultural belief is also a factor that can keep mothers from reporting to the hospital at the onset of diarrhea disease. Most of the rotavirus cases were identified in the age group 0-24 months and it was observed that rotavirus detection rate decreases with age. This is in accordance with other studies [9, 16] which also showed that rotavirus infection decreases with increasing age, due to acquired immunity that comes with age or asymptomatic infections [17, 15]. Overall, a seasonal trend was observed in this study with most of the rotavirus cases occurring in the dry season with a detectable peak in the months of November and December (Figure 2). This finding is in contrast to what was observed by Esona and co-workers in 2003 in Western Cameroon where rotavirus infection was observed all year round. Caution should be taken in interpreting these results as data was not collected for a sufficiently longer period.

Estimates on rotavirus mortality have been published previously. In 2003, Parashar and colleagues estimated ∼18,500 diarrhea and 3696 RVA-associated child mortality occur in Cameroon annually [18], whereas the WHO's 2004 report on child mortality due to diarrhea and rotavirus in Cameroon was ∼14,500 and 4369 deaths (95% CI, 3612-5126), respectively [19, 20]. These earlier estimates are based on lower detection rates (mean detection rate of 33%; 95% CI, 28-38) [4], for RVA utilized in calculations for Cameroon. Given the higher detection rate for RVA in this study, however, we anticipate that the number of rotavirus-associated deaths might be higher and stands at 6199 during 2010-2011 using the algorithm implemented by Parashar et al., (2003). This greater incidence of RVA infection is in agreement with estimates by the WHO for 2009-2010 [21].

## Conclusion

This study showed that the prevalence of rotavirus was high (42.8%) especially among children admitted to hospital due to diarrhea in Northern Cameroon. Seeing the increase in RVA detection rates, continuation of enhanced surveillance is needed to better understand rotavirus disease burden in Cameroon. Further characterizations of the positive RVA cases have been initiated.
